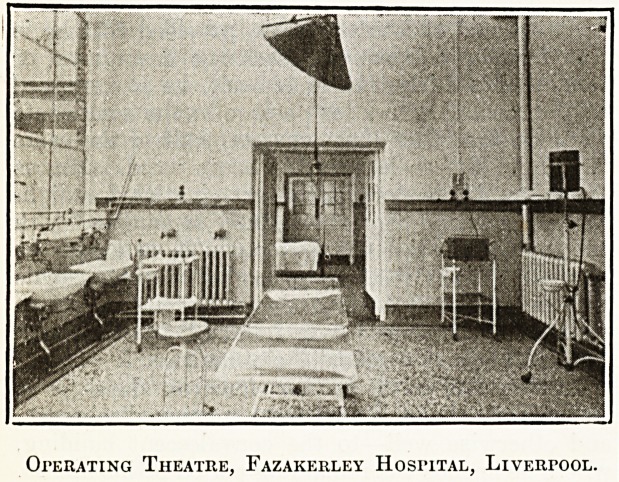# Preventive Methods in Provincial Institutions

**Published:** 1912-08-10

**Authors:** A. Knyvett Gordon

**Affiliations:** formerly Medical Superintendent of Monsall Hospital and Lecturer on Infectious Diseases in the University of Manchester.


### SOME FEVER HOSPITALS AND THEIR WORK.*

### III.—Preventive Methods in Provincial Institutions.

By A. KNYVETT GORDON, M.B. Cantab., formerly Medical Superintendent of Monsall Hospital and Lecturer on Infectious Diseases in the University of Manchester.

We now come to some further details in the administrative treatment of scarlet fever as this is carried out at the Leeds City Hospital under the direction of the Medical Superintendent, Dr. A. E. Pearson, to whose courtesy I am much indebted for information concerning the hospital.

On admission the patient is seen by one of the medical staff in one of two receiving rooms which are only used for cases of scarlet fever. The provision of two rooms obviates the difficulty which arises when a patient is suffering from some other infectious disorder; where there is only one room it is impossible to disinfect it before the next patient arrives. Personally, I am of the opinion that many more patients are infected in receiving rooms than is usually supposed, and for this reason I used always to examine patients in the ambulance, and they were not taken to a receiving room at all. Still, the risk with two rooms for scarlet fever is almost infinitesimal. The patient is then assigned to the appropriate ward as previously described, doubtful cases being sent to the scarlet fever isolation block, co-existent infections to another, and septic cases to be "barriered" in the acute ward.

The throats of the ordinary cases are treated by spraying and swabbing with antiseptic solutions, and syringing is not employed except for a few septic cases, when a ball syringe is used with a separate boiled nozzle for each patient, which is kept inside the barrier. Consequently the risk of infecting the ordinary cases by an unsterilisable appliance does not arise. Speaking of fever hospitals generally, there can be no doubt that much of the cross-infection that used to exist was caused by the use of one or two Higginsons syringes for the treatment of the throats of all, or nearly all, the patients in one ward.

### The Return Case.

We now come to the difficult problem of the "return case." After we have made a very necessary deduction for the cases that are not really return cases at all, but are due to insufficient disinfection of clothing and surroundings in the house from which the patient was removed—and for which, incidentally, the hospital often receives most undeserved blame—we have the fact that patients are occasionally discharged from fever hospitals who infect others after they have returned home.

Considering that we have no means of detecting by bacteriological examination the presence of the organism to which scarlet fever is due it is perhaps surprising that this does not happen much more frequently: at Leeds, for instance, during 1911 only 2.8 per cent. of the patients infected others subsequently. It seems to be almost certain that the explanation is that in hospital a patient has two infections: one, that with which he entered, and the other an extraneous infection derived from other patients in the same ward. In all probability most patients harbour (and incubate in their noses and throats) organisms which they have thus picked up from others, and if this be so, it is obvious that it is best not to discharge a child direct from the acute ward, but to employ a process of decantation, whereby the ordinary cases are transferred to convalescent wards where they are not exposed to the undoubtedly more virulent infection from the more acute cases.

### The Double Transfer System at Leeds.

The system in vogue at Leeds is as follows:—

The patient is admitted to one half of the acute ward, where he is protected from infection from septic cases, or those suffering from ringworm, etc., or who have been in contact previous to admission with other infections, such as measles or chicken-pox, by the careful "barriering" of all these latter.

At the end of fourteen days, provided that he is free from any infectious discharge from nose or ears, and is fairly well in himself, he is examined by the medical officer for passage to the semi-acute ward on the other (but separate) half of the same building. Before leaving the acute ward, cultures are taken for bacteriological examination for diphtheria bacilli, and his whole body is anointed with eucalyptus oil, and the nose, mouth, and ears are sprayed with a mixture of carbolic and salicylic acid solutions, and his scalp is thoroughly shampooed with antiseptic soap followed by inunction with sulphur and salicylic ointment.

At the end of a further fourteen days he is transferred—if he is free from mucous discharges, and otherwise well—to the convalescent building, where he spends the remaining period of his illness. Cultures are taken on leaving the semi-acute ward, and again before discharge from hospital.

When the patient has been passed—as free from visible infection—by the medical officer, and is ready to leave the hospital, his clothes are disinfected by steam, and he goes wrapped up in blankets to the discharge block, where the nurse and porter on duty are not employed in any other wards. He is then dressed in his clean clothes and handed over to the care of his friends, who are warned of the risk of infection in cases where there has been any previous septic complication.

This process of double transfer is excellent, and eliminates as far as possible the risk of a patient taking his own or other people's infection away.

Another point that tends to diminish the risk of cross-infection at Seacroft is the fact that the ambulances in which patients are removed to hospital are not staffed by nurses who have to be taken from the wards for the purpose. When one comes to think of it, the contact between the patient and the nurse inside an ambulance is very intimate,

* Previous articles appeared in The Hospital of July 13 and 20.

far more so than occurs under ordinary circumstances in the wards, and given that any patient who has been removed in an ambulance by a nurse taken from a scarlet fever ward is suffering from measles, for instance, it is very difficult to ensure—even with the best intentions—that the nurse and her clothing are efficiently disinfected before she enters the ward again. In times of pressure this is apt to be hurried.

Great attention is paid at Seacroft to the training of the nurses: probationers are accepted for training for a definite period only, and they receive lectures during their first year on anatomy and physiology, and during the second year on the principles of infection and on the various infectious diseases; an examination, conducted by an outside examiner, is held at the conclusion of each course. The daily average of nurses (including sisters) is 84.5 for an average of 392 patients, there being thus one nurse to 4.6 patients. Considering the care which is taken with the details of asepsis, this proportion would appear to be none too high, and the margin for accidents in the shape of illness and absence amongst the nurses is rather small.

Operating Theatre, Fazakerley Hospital, Liverpool.

In the hospital laboratory nearly eleven thousand bacteriological examinations were made during 1911, which illustrates the scientific nature of the work.

I shall have occasion to refer to the Leeds Hospital again when we come to consider the treatment of diphtheria and enteric fever, but we will now deal with scarlet fever in some other institutions.

### The New Hospital at Liverpool.

We will take next the new hospital erected by the Corporation of Liverpool at Fazakerley. The buildings here were constructed by the City Surveyor, and are well adapted for the aseptic technique which is in vogue there. The most interesting feature of the work is the treatment of phthisis on sanatorium lines, to which great attention is paid by the city authorities, but which is outside the scope of these articles. In addition, cases of scarlet fever, diphtheria, and measles are treated in bulk, and also a few patients suffering from erysipelas and puerperal fever. In summer about sixty beds are devoted to cases of epidemic diarrhœa. The total number of beds is 510.

With such a varied assortment of diseases under treatment it will be obvious that the risks of cross-infection are great. The admission of measles, in particular, adds considerably to the difficulties of administration, for this disease is not only so highly infectious as often to necessitate the separation of the nurses who have the care of the cases from the rest of the staff even when off duty, but is often fatal to convalescents from diphtheria or scarlet fever.

At Fazakerley there are seventy-two isolation beds out of a total of 510, and though this proportion is fairly high, it does not make structural separation of every doubtful or highly infectious case possible. Consequently, great reliance has to be placed on the details of aseptic technique as regards the nursing, and a system of bed isolation has been evolved by the Medical Superintendent, Dr. C. Rundle, which has given really excellent results; I am indebted for the details of this to a paper by himself and Dr. A. H. G. Burton in the *Lancet* of March 16, 1912.

### Throat Treatment at Fazakerley.

Before dwelling further on this, it will be well to mention one or two points in the general treatment of scarlet fever. At Fazakerley neither syringing, spraying, nor douching is ever employed in the treatment of the throats, but reliance is placed on swabbing with antiseptic solutions instead. Normal horse serum is given (subcutaneously) to a large proportion of the severe cases, which are also treated whenever possible in the open air. The serum probably acts by causing a leucocytosis, in much the same way as the injection of normal saline solution, which often acts like a charm in septic cases.

Convalescent cases are transferred once and for all either to a special ward or to another hospital as occasion serves. On discharge from hospital, ordinary cases are bathed and dressed in the discharge block on the day on which they leave the hospital. Those who still have mucous discharges are bathed the day before, and placed in an uninfected ward for twenty-four hours, and this type of patient is always isolated from others whenever the accommodation permits for three or four days previously. All patients are examined bacteriologically for the presence of diphtheria bacilli before leaving hospital. There is a large and well-appointed laboratory in which all internal bacteriological work is carried out by the medical staff of the hospital.

It will now be convenient to describe the method of bed isolation referred to. This is carried out in one pavilion only, which contains twenty-three beds and is divided into a male and female side, which are separated by the ward kitchen. Each half has a small single-bedded separation ward opening out of it, and that on the male side leads into a modern operating theatre, which is here illustrated. To this pavilion are admitted (1) all cases of puerperal fever and erysipelas; (2) most cases of whooping-cough, rubella, and chicken-pox; (3) all cases notified as scarlet fever or diphtheria, but found on admission not to be suffering from any infectious disease at all; (4) doubtful cases of scarlet fever and diphtheria; (5) cases from other (infectious) wards requiring operative treatment; (6) convalescent cases of diphtheria and measles; (7) epidemic diarrhœa.

No Structural Separation.

The point about the system is that there is no structural separation whatever, but that all these different types of cases are placed in any position that may be convenient in the ward; the beds are not even marked or surrounded by screens. The precautions taken are of two degrees. For patients in classes (1), (3), and (7) these consist in the observance of surgical cleanliness only, with the use of rubber gloves for the surgeon and nurse wherever necessary; coats are worn both by the surgeon and nurse. For the rest, namely, those patients who have, or may have an infectious disease of some kind or other, all utensils are boiled after use and kept separate for each patient. Coats are worn, and both the surgeon and nurse wash their hands in disinfectant solution before leaving the bedside. Any patient judged to be infectious is not allowed up until he is free from infection, but the others are permitted to leave their bed when they are physically fit to do so, and then to mix with other non-infectious cases.

During the past two years 554 patients were admitted to the pavilion suffering from infectious diseases—namely, scarlet fever, diphtheria, measles, rubella, varicella, erysipelas, typhoid, and puerperal fevers—and a further 114 had no infectious illness at all. Only two patients contracted any infection whilst under treatment—a result which is exceptionally good. The reason for this brilliant success is undoubtedly to be found in the fact that the nursing staff in the pavilion were exceptionally numerous and well trained. In charge of the whole block is a sister with special surgical and fever experience, and there is on each side a staff nurse with general and fever training, and also one probationer. At night there are two nurses, one of whom has had three years' general training in addition to fever experience. The results show that the question of infection by aërial convection can be ignored, and that, therefore, structural separation of the patients is not necessary.

It has yet to be shown, however, that the method is applicable to all of the general wards, unless the same number and quality of nurses could be obtained, and for them I prefer the "barrier" system.

## Figures and Tables

**Figure f1:**